# Integrated analysis of single-cell and bulk RNA sequencing data reveals a cellular senescence-related signature in hepatocellular carcinoma

**DOI:** 10.3389/fcell.2024.1407428

**Published:** 2024-06-03

**Authors:** Lei Qiao, Zibo Xu, Yuheng Chen, Wenwei Chen, Yuan Liang, Yi Wei, Kang Wang, Yue Yu, Wei Yan

**Affiliations:** ^1^ Hepatobiliary Center, The First Affiliated Hospital of Nanjing Medical University, Key Laboratory of Liver Transplantation, Chinese Academy of Medical Sciences, NHC Key Laboratory of Living Donor Liver Transplantation (Nanjing Medical University), Jiangsu Provincial Medical Innovation Center, Jiangsu Provincial Medical Key Laboratory, Nanjing, Jiangsu Province, China; ^2^ Department of Bioinformatics, Nanjing Medical University, Nanjing, China; ^3^ School of Public Health, Southeast University, Nanjing, Jiangsu Province, China; ^4^ School of Biological Science and Medical Engineering, Southeast University, Nanjing, Jiangsu Province, China; ^5^ Department of Burn and Plastic Surgery, The First Affiliated Hospital of Nanjing Medical University, Nanjing, Jiangsu Province, China

**Keywords:** cellular senescence, hepatocellular carcinoma, prognosis, tumor immune microenvironment, treg

## Abstract

The mortality of hepatocellular carcinoma (HCC) is on the rise globally, particularly in the Western world, with etiology gradually shifting from virus-related liver diseases to metabolic disorders such as non-alcoholic fatty liver disease. Early detection of HCC is challenging, and effective prognostic indicators are currently lacking, urgently necessitating reliable markers to assist in treatment planning and clinical management. Here, we introduce hepatocellular carcinoma senescence genes (HSG) to assess cellular senescence in HCC and devise a hepatocellular carcinoma senescence score (HSS) for prognostic prediction. Higher HSS levels signify poorer prognosis and increased tumor proliferation activity. Additionally, we observe alterations in the tumor immune microenvironment with higher HSS levels, such as increased infiltration of Treg, potentially providing a basis for immunotherapy. Furthermore, we identify key genes, such as PTTG1, within the senescence gene set and demonstrate their regulatory roles in HCC cells and Treg through experimentation. In summary, we establish a scoring system based on hepatocellular carcinoma senescence genes for prognostic prediction in HCC, potentially offering guidance for clinical treatment planning.

## 1 Introduction

Hepatocellular carcinoma (HCC) is one of the most common malignant tumors worldwide. Following the GLOBOCAN 2020 database, primary liver cancer is the sixth most commonly diagnosed cancer and the third leading cause of cancer death worldwide in 2020, with approximately 906,000 new cases and 830,000 deaths (Sung et al., 2021). China is one of the most high-risk HCC areas with about 393,000 new cases of liver cancer and up to 369,000 deaths per year, accounting for more than 50% of the global liver cancer (European Association for the Study of the LiverEuropean Association for the Study of the Liver, 2018). Although hepatectomy and liver transplantation are the most effective treatments for HCC, tumors often recur and metastasize via the bloodstream following surgery. From sorafenib, the first molecularly-targeted drug approved by the FDA for the treatment of advanced HCC in 2007, to regorafenib and Lenvatinib, the median survival in the treatment group was only prolonged by 3–4.3 months. Overall survival (OS) and 5-year survival in HCC patients remain disappointing, with efficacy lagging behind that of most other oncological diseases (Agarwal et al., 2017; Allemani et al., 2018; Kudo et al., 2018; Cao et al., 2020). Therefore, it is imperative to identify new therapeutic biomarkers to understand and overcome HCC.

Cellular senescence refers to a physiological status of cell cycle arrest in response to endogenous and exogenous stress, characterized by persistently ceased proliferation but retained metabolic activity, so tissues with renewable properties have been reported to be more vulnerable (Jeyapalan et al., 2007; Calcinotto et al., 2019). Moreover, chronic inflammation has been strongly linked to cell senescence (Chung et al., 2019). In China, the key risk factors of HCC are chronic HBV infection, aflatoxin exposure, or both, which means hepatocellular carcinoma is often accompanied by chronic liver inflammation and cirrhosis (Sung et al., 2021; Zhang et al., 2023). A growing number of research has shed light on the dichotomous role of cellular senescence in HCC progression. Cellular senescence can activate immunosurveillance to eliminate or kill HCC cells in various ways at early stages (Campisi, 2001; Wang et al., 2022). However, persistent senescent cells tend to modify the tumor microenvironment through the senescence-associated secretory phenotype (SASP), thus activating immunosuppression, boosting cell proliferation, driving tumor vascularization, and favoring tumor progression (Calcinotto et al., 2019; Lau and David, 2019; Prieto and Baker, 2019; Xiong et al., 2023). Therefore, the senescence-related genes may be a potential index for predicting the prognosis of patients with HCC.

Compared to other tissues and cancer models, the role of senescence in liver cells and its implications in hepatocellular carcinogenesis have been less explored. However, the development of high-throughput detection techniques and big data resources provides a method for the connection between senescence-related genes and prognosis of HCC. In this study, we proposed a hepatocellular carcinoma senescence score (HSS) composed of multiple hepatocellular carcinoma senescence genes (HSG) and constructed a risk model to predict the prognosis of (HCC) patients. Additionally, we further classified the immune status, immune function, and cancer cell mutation spectrum of the high HSS group and low HSS group, dissecting the reasons for the poor prognosis of the high HSS group. Furthermore, focusing on the key gene PTTG1 in HSG, we investigated its functions in hepatocellular carcinoma cells and Treg. These findings elucidate the complex interaction between hepatocellular carcinoma and cellular aging, providing a basis for the future development of therapeutic strategies targeting cellular aging in hepatocellular carcinoma.

## 2 Materials and methods

### 2.1 Collection of bulk RNA expression datasets

The hepatocellular carcinoma bulk RNA sequencing datasets analyzed in this study were retrieved from the HCCDB v2.0 (http://lifeome.net/database/hccdb2) database and consisted of 756 patients from 4 separate datasets. Of these, the Cancer Genome Atlas (TCGA) dataset comprised 347 patients, the International Cancer Genome Consortium (ICGC) dataset had 203 patients, the expression profile of OEP000321 had 158 patients, and 48 patients from the Gene Expression Omnibus (GEO) database (GSE148355). Log2 transformation was applied to normalized read counts from those bulk RNA sequencing datasets and some patients with missing data on survival status or gene expression data were excluded. In Integrated dataset cohorts, we used the limmaremoveBatchEffect package to correct batch effects from different datasets. The basic information of these datasets is shown in Table 1.

**TABLE 1:** Clinical information table.

Characteristic	Total [756]	GSE148355 [48 (6.3%)]	ICGC [203 (27%)]	OEP000321 [158 (21%)]	TCGA [347 (46%)]
Gender					
Female	194 (26%)	7 (15%)	50 (25%)	30 (19%)	112 (32%)
Male	557 (74%)	41 (85%)	153 (75%)	128 (81%)	235 (68%)
Age					
Mean (SD)	60 (13)	55 (10)	67 (10)	54 (11)	60 (13)
Median (IQR)	61 (52, 69)	55 (49, 60)	69 (62, 74)	54 (46, 62)	61 (52, 69)
Range	17, 90	36, 78	31, 86	20, 81	17, 90
Stage					
Other	24 (3.2%)	0 (0%)	0 (0%)	0 (0%)	24 (6.9%)
Stage I	301 (40%)	15 (31%)	33 (16%)	90 (57%)	163 (47%)
Stage II	209 (28%)	21 (44%)	96 (47%)	14 (8.9%)	78 (22%)
Stage III	199 (26%)	9 (19%)	59 (29%)	52 (33%)	79 (23%)
Stage IV	23 (3.0%)	3 (6.3%)	15 (7.4%)	2 (1.3%)	3 (0.9%)
Status					
Alive	531 (70%)	42 (88%)	168 (83%)	102 (65%)	219 (63%)
Dead	225 (30%)	6 (13%)	35 (17%)	56 (35%)	128 (37%)
Virus					
HBV	180 (24%)	36 (75%)	53 (26%)	0 (0%)	91 (26%)
HBV,HCV	11 (1.5%)	0 (0%)	4 (2.0%)	0 (0%)	7 (2.1%)
HCV	168 (22%)	5 (10%)	117 (58%)	0 (0%)	46 (13%)
Other	397 (52%)	7 (15%)	29 (14%)	158 (100%)	203 (58%)

HBV, hepatitis B virus; HCV, hepatitis C virus.

### 2.2 Single-cell data source and preprocessing

The single-cell RNA sequencing (scRNA-seq) data of hepatocellular carcinoma in GSE149614 and GSE156625 were obtained from the NCBI (Gene Expression Omnibus (https://www.ncbi.nlm.nih.gov/geo/). Gene expression data for individual samples were analyzed using Read10×() in the Seurat [25867923] package (v5.0.1) of R software (v4.3.1). We processed the dataset GSE149614 using the Seurat R package, low-quality cells with ≤200 detected genes or ≥20% mitochondrial genes were removed. The dataset GSE149614 using the Seurat R package, low-quality cells with ≤200 detected genes or ≥5% mitochondrial genes were removed. Merge datasets GSE149614 and GSE156625, excluding samples with fewer than 1,000 cells and normalized by NormalizeData, and the top 4,000 highly variable genes were subsequently identified by FindVariableFeatures (). Principal Component Analysis (PCA) was performed using the RunPCA function is used for data dimensionality reduction. Batch effects were removed from all samples using the Harmony package (v1.2.0) (Korsunsky et al., 2019). K-nearest neighbors were calculated using Harmony-corrected data, followed by the creation of a shared nearest neighbor (SNN) plot. Additionally, the first 50 principal components (PCs) were selected for downstream analysis and clustered the cells using the FindClusters function, setting the Resolution parameter to 1. Using the -distributed Stochastic Neighbor Embedding (tSNE) dimensionality reduction technique, the identified clusters were visualized on a 2D map.

### 2.3 Construction of a prognostic senescence signature

We consulted the CellAge database (https://genomics.senescence.info/cells/cellAge.zip), which comprises 949 cellular senescence genes. Additionally, we retrieved pathways related to Cellular_senescence from the Kyoto Encyclopedia of Genes and Genomes (KEGG) and Gene Ontology (GO) databases, obtaining two additional gene sets. Upon merging with the CellAge data, we obtained a total of 996 genes related to cellular senescence (Supplementary Table S1).

To identify differentially expressed genes (DEGs) between tumor and adjacent normal tissues, we conducted differential analysis on dataset samples utilizing the limma package (version 3.58.1). The established thresholds were set at *p*-value <0.05 and |logFC|>1. Univariate Cox approach using the survival package to identify the gene set for cellular senescence associated with prognosis. *p* < 0.05 and HR > 1 in the univariable Cox regression analysis were considered statistically significant. Principal Component Analysis (PCA) is used to demonstrate batch effects among the data. We then utilized the glmnet R package to perform the least absolute shrinkage and selection operator (LASSO) regression of the prognostic genes. Then, four genes were identified and used to create a risk score using the GSVA R package (Hanzelmann et al., 2013). The survival curves were created by Kaplan–Meier (KM) analysis and the log rank test using the survival R package to assess the accuracy of the prediction (Sun et al., 2024), and ROC curves for the risk scores were constructed using the timeROC R package.

### 2.4 Immune infiltration analysis

The CIBERSORT algorithm was utilized to analyze transcriptome data and obtain the expression levels of 22 types of immune cells in each sample (Chen et al., 2018). The quanTIseq algorithm was utilized to analyze transcriptome data and obtain the expression levels of 11 types of immune cells in each sample (Finotello et al., 2019). The ESTIMATE package was used to analyze the composition of the tumor microenvironment (Yoshihara et al., 2013). We also calculated the Immunophenoscore (IPS) for each sample to assess four different immunophenotypes separately (Charoentong et al., 2017). Scoring of four types of relevant molecules, Including MHC molecules (MHC); Checkpoints| lmmunomodulators (CP); Effector cells molecules (EC); Suppressor cells molecules (SC). AZ, the sum of scores for four types; Immunophenoscore (IPS), was calculated on an arbitrary 0–10 scale based on the sum of the weighted averaged Z score of the four categories.

### 2.5 Gene set enrichment analysis

In order to shed further light on the biological processes (BP), cellular components (CC), molecular functions (MF), and pathways involved with DEGs. The R ClusterProfiler tool analyzed DEGs using Gene Ontology (GO) and the Kyoto Encyclopedia of Genes and Genomes (KEGG) (Yu et al., 2012). The Gene Set Enrichment Analysis (GSEA) (Subramanian et al., 2005) method is also used to explore the biological functions of DEGs. We also calculated the pathway activities of tumor samples using the GSVA R package. The gene-signatures included for analysis were downloaded from Hallmark gene sets and C2 curated gene sets (MSigDB database v7.5.1) (Liberzon et al., 2011). Hallmark pathway enrichment analysis is implemented using the enricher tool.

### 2.6 Somatic mutation analysis

The somatic mutation data (TCGALIHC) were downloaded from Genomic Data Commons using the TCGAmutations R package. The Maftools R package was applied to analyze the mutation annotation format (MAF) from TCGA and was used to identify mutant genes and calculate TMB level (Mayakonda et al., 2018).

### 2.7 Identification of marker genes for cell clusters

To identify the marker genes for each one of those 40 cell clusters, we contrasted cells from a cluster to all the other cells of that cluster using the FindMarkers function of Seurat, which identifies differentially expressed genes between two groups of cells using a Wilcoxon rank-sum test. Classical cell-specific marker genes also were used to identify cell types.

### 2.8 Assessment of cell states

The score of each cell’s senescence prognosis genes in single-cell transcriptome data is calculated using Aucell. AUCell is an R-package to analyze the state of gene sets in single-cell RNA-seq data. We gathered a classic cell proliferation gene set (Supplementary Table S2) and evaluated the proliferative capacity of cells using Aucell. Additionally, we utilized the CytoTRACE (Gulati et al., 2020) R package (version 0.3.3) to aid in predicting the direction of cell differentiation. Calculations were performed with default parameters using the R package infercnv (v1.19.1) with annotated T cells as a reference, and clusters were reassigned to malignant cells via the CNV matrix in Hepatocyte. The CNV score is calculated by multiplying the mean of the normalized CNV matrix in each cell by 100. Furthermore, we calculated the correlation between the CNV matrix of each cell and the CNV score of the top 10% ranked cells using the Pearson coefficient.

### 2.9 Metabolic analysis

scMetabolism (v0.2.1) is an R package suitable for quantification of cellular metabolic activity at single-cell resolution (Wu et al., 2022). scMetabolism was applied to analyze the activation of KEGG metabolic pathways in hepatocytes. The metabolic pathway activity of 3 cell types in Hepatocyte was represented by the average activity score of metabolic pathways.

### 2.10 SCENIC analysis

SCENIC was used to identify the shared regulatory networks by utilizing the putative regulatory binding sites found in promoter regions (Van de Sande et al., 2020). To investigate the transcription factor (TF) activity in single cells, SCENIC analysis was run using the SCENIC packages in R software (v4.3.1) and pySCENIC in Python (v 3.6.6). The activity of these regulons is quantified via an enrichment score for the regulon’s target genes (AUCell). The philentropy R package utilizes Jensen-Shannon divergence to compute dissimilarity, while ggplot is used to visualize the specificity activation scores of each regulon in 3 cell types of Hepatocyte. Finally, the protein-protein interaction (PPI) network was constructed using the STRING (https://string-db.org/) database with target genes and transcription factors (TFs).

### 2.11 Cell-cell communication analysis

We utilized CellChat, a tool that quantitatively infers intercellular communication networks from scRNA-seq data (Jin et al., 2021). Based on a database of human ligand-receptor interactions and pattern recognition techniques, CellChat can detect intercellular communication at the pathway level and calculate the communication network of aggregated cells. Use default settings for all parameters.

### 2.12 Cell culture

The Huh7 human hepatocellular carcinoma cell line was purchased from American. Type Culture Collection. The cells were cultured in Dulbecco’s Modified Eagle Medium (DMEM) supplemented with 10% fetal bovine serum (FBS) and 1% penicillin-streptomycin.

The extraction and cultivation of human Treg cells were performed following previously reported methods (Gu et al., 2022). In brief, peripheral blood cells from volunteers at Nanjing Medical University were extracted, and naïve T cells were obtained through magnetic bead selection. Subsequently, these cells were induced into iTreg *in vitro* using IL-2 and TGF-β.

### 2.13 Lentivirus transfection

The generated lentivirus was used to knock down PTTG1 in both Huh7 and iTreg. In Huh7 cells, on the second day of subculture, 100 μL of lentiviral supernatant was added to 2 mL of complete medium. After infecting the cells with the lentivirus for 10 h, the supernatant was removed and replaced with 2 mL of fresh complete medium. The infection efficiency was observed 48 h later. After confirming the successful transfection of the lentivirus into Huh7 cells, cells were selected with a complete medium containing 0.2 μg/mL puromycin for 7 days. After 7 days of selection, cells were returned to a puromycin-free complete medium for further cultivation. In iTreg, iTreg induction was conducted in 6-well plates. 16 h after the induction of iTreg, lentiviral supernatant was added to the cells. The cells were then centrifuged at 200 *g* for 90 min and cultured at 37°C for 72 h, followed by flow cytometry sorting to obtain successfully transfected cells.

### 2.14 EdU assay

Following the manufacturer’s instructions, an EdU assay was conducted utilizing an EdU Cell Proliferation Kit (Biosharp, China). Briefly, cells were cultured in a 6-well plate at a density of 3 × 10^6 cells per well and treated with 10 μM EdU per well. Subsequently, the cells were fixed with 4% paraformaldehyde and stained using 1× click reaction buffer and 1× DAPI solution. Lastly, cell counting was performed under a Zeiss fluorescence photomicroscope.

### 2.15 Colony formation assay

Transfected Huh7 cells were plated into 6-well plates at a density of 1,000 cells per well and incubated for 2 weeks. Subsequently, the cells were fixed and stained in a dye solution comprising 0.1% crystal violet (Beyotime, China) and 100% methanol.

### 2.16 Immunohistochemical (IHC) staining

We first deparaffinized and rehydrated tissue sections (4 µm). Subsequently, endogenous peroxidase activity was blocked using 3% hydrogen peroxide. The sections were then subjected to antigen retrieval in EDTA buffer (pH 9.0) at 96°C for 20 min. Following antigen retrieval, the sections were incubated with a PTTG1 primary antibody (Affinity, af0354) overnight at 4°C. They were subsequently incubated with secondary antibodies for 30 min at 37°C and stained with 3,3-diaminobenzidine. All procedures were carried out in accordance with the manufacturer’s instructions.

### 2.17 RNA extraction and quantitative polymerase chain reaction (qPCR)

Total RNA was extracted from cells with an RNA-Qucik Purification Kit (Yishan, Shanghai, China). Reverse transcription was performed with the HiScript II 1st Strand cDNA Synthesis Kit (Vazyme, Nanjing, China) according to the manufacturer’s instructions. qPCR was used to detect gene expression with AceQ universal SYBR qPCR Master Mix (Vazyme, Nanjing, China). Results were normalized against Gapdh expression respectively. The expression levels were calculated by the 2˗ΔΔCT method. The primers used for the amplification were shown in Supplementary Table S4.

### 2.18 Statistical analysis

All statistical analyses were calculated using R (v4.1.0) and Python (v 3.6.6). Based on the recommended methods, the statistical analyses of different datasets were performed using different packages. A significance threshold of *p* < 0.05 was applied to determine statistical significance. The Spearman correlation coefficient was used to characterize the correlation, and a *p*-value less than 0.05 was considered statistically significant. Asterisks are used to indicate statistical significance (*, *p* < 0.05; **, *p* < 0.01; ***, *p* < 0.001); ns, there was no statistical significance (*p* > 0.05).

## 3 Results

### 3.1 Identification of Prognosis-Associated Genes Related to hepatocellular carcinoma senescence

**FIGURE 1:**
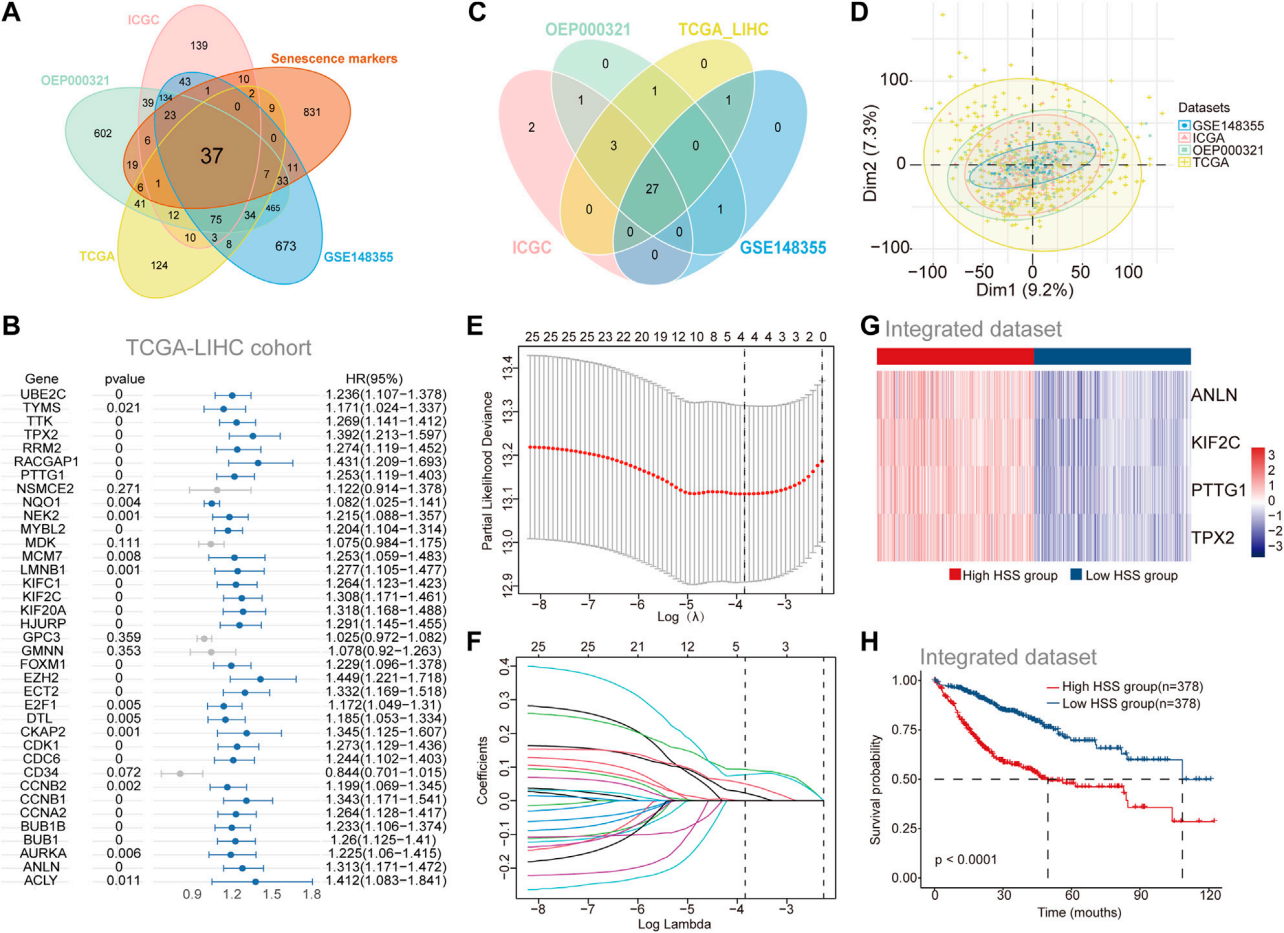
Identification of Prognosis-Associated Genes Related to Hepatocellular Carcinoma Senescence. **(A)** The Venn diagram shows the overlap between differential genes in Tumor *versus* adjacent normal tissues and Senescence markers. **(B)** Univariate Cox regression of differential genes for the screening of prognosis-related genes in TCGA-LIHC cohort. **(C)** The Venn diagram shows the overlap results of Univariate Cox regression analysis of the 4 separate datasets (genes with HR > 1, *p* < 0.05). **(D)** Principal component analysis (PCA) of the 4 separate datasets with Batch_limma. **(E–F)** Least absolute shrinkage and selection operator (LASSO)further screens for genes associated with prognosis. **(G)** Gene expression of High HSS group and Low HSS group. **(H)** Kaplan–Meier curves based on the High/Low HSS group in the Integrated dataset-OS.

To investigate the role of aging gene sets in the progression of HCC, we utilized four independent datasets from HCCDB v2.0. Differential expression analysis between tumor and adjacent non-tumor tissues yielded four sets of differentially expressed gene sets (Supplementary Figure S1A). The intersection of these upregulated genes in tumor tissues with a previously collected cellular senescence set identified 37 differentially expressed genes associated with cellular senescence (Figure 1A). Subsequently, to further elucidate the relationship between the cellular senescence set and prognosis of HCC patients, we combined patient survival data with expression profile data and employed univariate COX regression analysis to screen for genes significantly correlated with prognosis across the four datasets (Figures 1B, C; Supplementary Figure S1B-D), resulting in a total of 27 genes associated with prognosis. While incorporating as many genes as possible enhances the accuracy of constructing a prognostic risk model, our aim was to select a subset of stable genes. The four datasets prior to batch correction exhibited noticeable batch effects (Supplementary Figure S1E), which were effectively mitigated using the “Batch_limma” package (Figure 1D). Lasso regression was then applied to optimize the aforementioned gene sets in the integrated data to improve the efficiency of the prognostic risk model (Figures 1E, F), ultimately yielding four stable genes (PTTG1, ANLN, KIF2C, TPX2) for establishing the prognostic risk model. Employing the “GSVA” scoring method, hepatocellular carcinoma patients were stratified into high HSS and low HSS groups based on the median score, revealing significant differences in gene expression between the two groups (Figure 1G). Principal component analysis (PCA) results also demonstrated that the two patient groups did not belong to the same category (Supplementary Figure S1F). Combining patient survival data, Kaplan-Meier curve analysis revealed a significantly poorer prognosis in the high HSS group (Figure 1H). Finally, we assessed the predictive ability of the risk model, and time-dependent ROC analysis demonstrated the strong predictive capability of the constructed risk model at 1 year (Supplementary Figure S1G). In summary, we found that HSG (PTTG1, ANLN, KIF2C, TPX2) was significantly upregulated in hepatocellular carcinoma and that the constructed prognostic risk model can reliably predict the prognosis of hepatocellular carcinoma patients.

### 3.2 Analysis of Immune Status and Function in High and Low HSS groups

**FIGURE 2:**
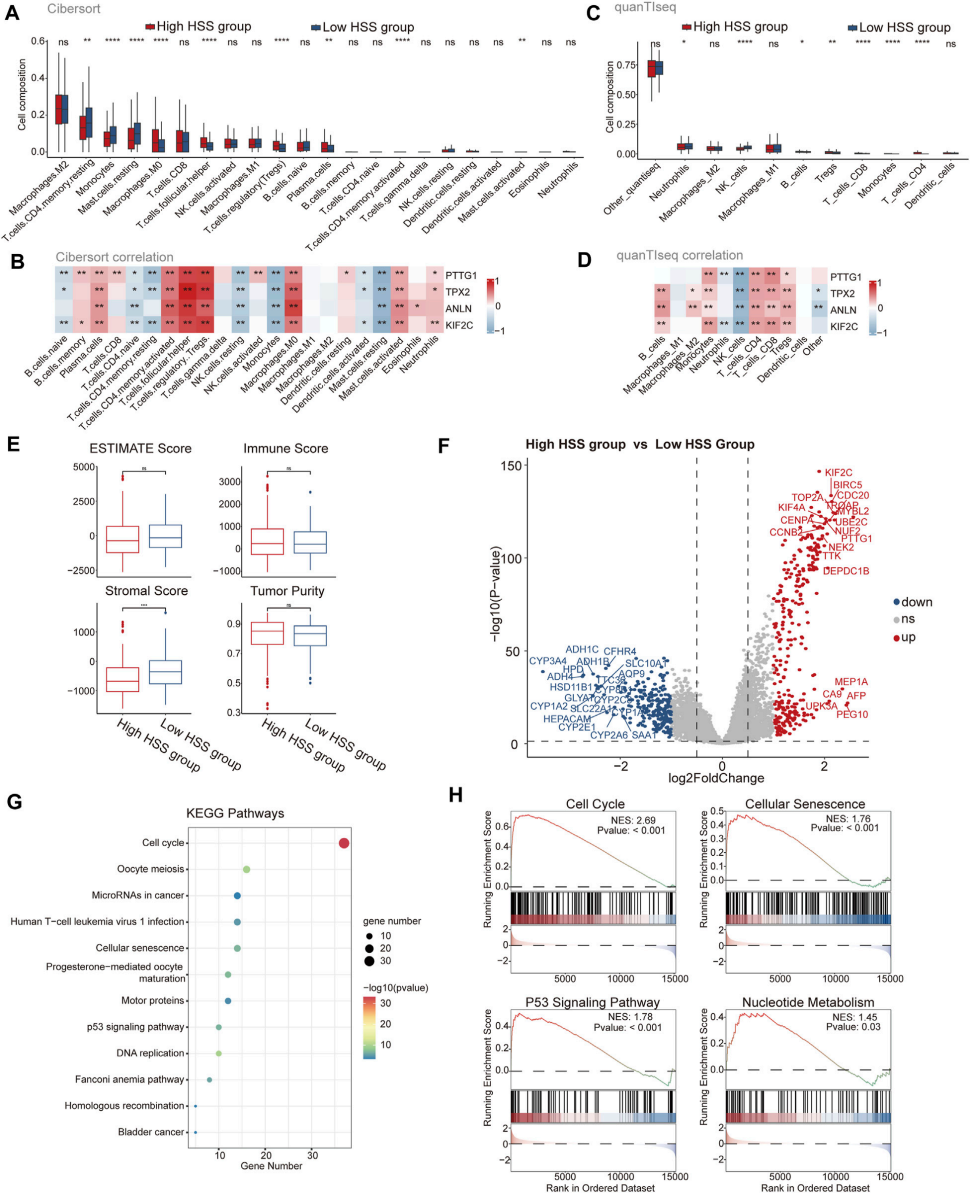
Analysis of Immune Status and Function in High and Low HSS Groups. **(A)** The subpopulation of infiltrating immune cells by CIBERSORT. **(B)** The correlation between gene expression and infiltration levels of 22 types of immune cells. **(C)** The subpopulation of infiltrating immune cells by quanTIseq. **(D)** The correlation between gene expression and infiltration levels of 11 types of immune cells. **(E)** Tumor purity, ESTIMATE, immune and stromal score of two groups in the Integrated dataset. **(F)** Differential expression analysis of High HSS group and Low HSS Group in the Integrated dataset. (*p*-value< 0.05 and log2FC > 1) **(G)** KEGG enrichment analysis of the up signature genes in the High HSS group. **(H)** Gene set enrichment analysis (GSEA) of the up signature genes in the High HSS group. (*p < 0.05; **p < 0.01; ***p < 0.001).

To further assess the differences in the immune microenvironment between the high and low HSS groups in HCC, we evaluated the levels of immune cell infiltration in the integrated dataset using gene expression levels of immune cells. Initially, we employed the CIBERSORT algorithm to determine the relative abundance of 22 immune cell types, revealing significant differences between the two patient groups associated with nine immune cell types, including Treg and macrophages (Figure 2A). Additionally, we assessed the correlation between HSG and the infiltration levels of 22 immune cells, finding that the infiltration level of each cell type was associated with at least one gene, except for M1 and M2 macrophages (Figure 2B). Due to potential differences in immune cell types and genes across different databases, we also utilized the quanTIseq algorithm to assess the immune infiltration levels of different cell types. We found higher infiltration levels of Treg in the high HSS group, and all four genes of HSG were positively correlated with Treg infiltration levels (Figures 2C, D), consistent with the results from the CIBERSORT algorithm. Furthermore, we employed the ESTIMATE algorithm to quantify the overall immune infiltration level (immune score) of immune cells, while also calculating the stromal score and tumor purity of the samples (Figure 2E). The results indicated slightly higher tumor purity in the high HSS group compared to the low HSS group. In addition to immune cells, we focused on the expression of relevant immune factors in the tumor immune microenvironment, combining the immunophenoscore (IPS) algorithm to assess the immune phenotype of tumor samples, including Antigen Processing-related immune factors, Effector Cells-related immune factors, Suppressor Cells-related immune factors, and Checkpoints-related immune factors. Significant differences were observed between the high and low HSS groups in four relevant immune factors, with the low HSS group showing higher expression of Antigen Processing-related molecules and a higher IPS (Supplementary Figure S2 A).

Differential expression analysis of the high/low HSS groups revealed significant upregulation of genes such as PTTG1 (Figure 2F). Subsequently, we performed KEGG and GO enrichment analyses on the upregulated gene set, revealing significant enrichment of pathways related to cell cycle and cellular senescence in the KEGG results (Figure 2G). Similarly, GSEA analysis also yielded similar results (Figure 2H), but also identified pathways related to nucleotide metabolism, consistent with the significant enrichment of DNA and chromosome-related biological activities in the GO enrichment results (Supplementary Figure S2 B). Finally, we utilized the classic HALLMARK pathways of tumors to score the high/low HSS groups, revealing minor overall differences but showing higher activity in cell proliferation-related pathways in the high HSS group, such as the HALLMARK_E2F pathway (Supplementary Figure S2C). In conclusion, significant differences exist in the tumor microenvironment of high/low HSS groups in hepatocellular carcinoma, with the high HSS group exhibiting increased activity in cell proliferation-related pathways.

### 3.3 Study of Hepatocellular Carcinoma Mutation Spectrum

Based on the content of the above results, differences were found in the IPS_IPS scores between the high and low HSS groups. Relevant literature suggests that a higher immune phenotype score corresponds to a better response to immune checkpoint inhibitors. Additionally, there are reports indicating that tumor mutation burden (TMB) is also an important factor influencing immune checkpoint inhibition. Therefore, we downloaded mutation expression profiles of TCGA data and combined them with previously analyzed transcriptome expression profiles, resulting in a total of 336 patients with retained mutation and expression data. We conducted separate analyses for the high and low HSS groups.

**FIGURE 3:**
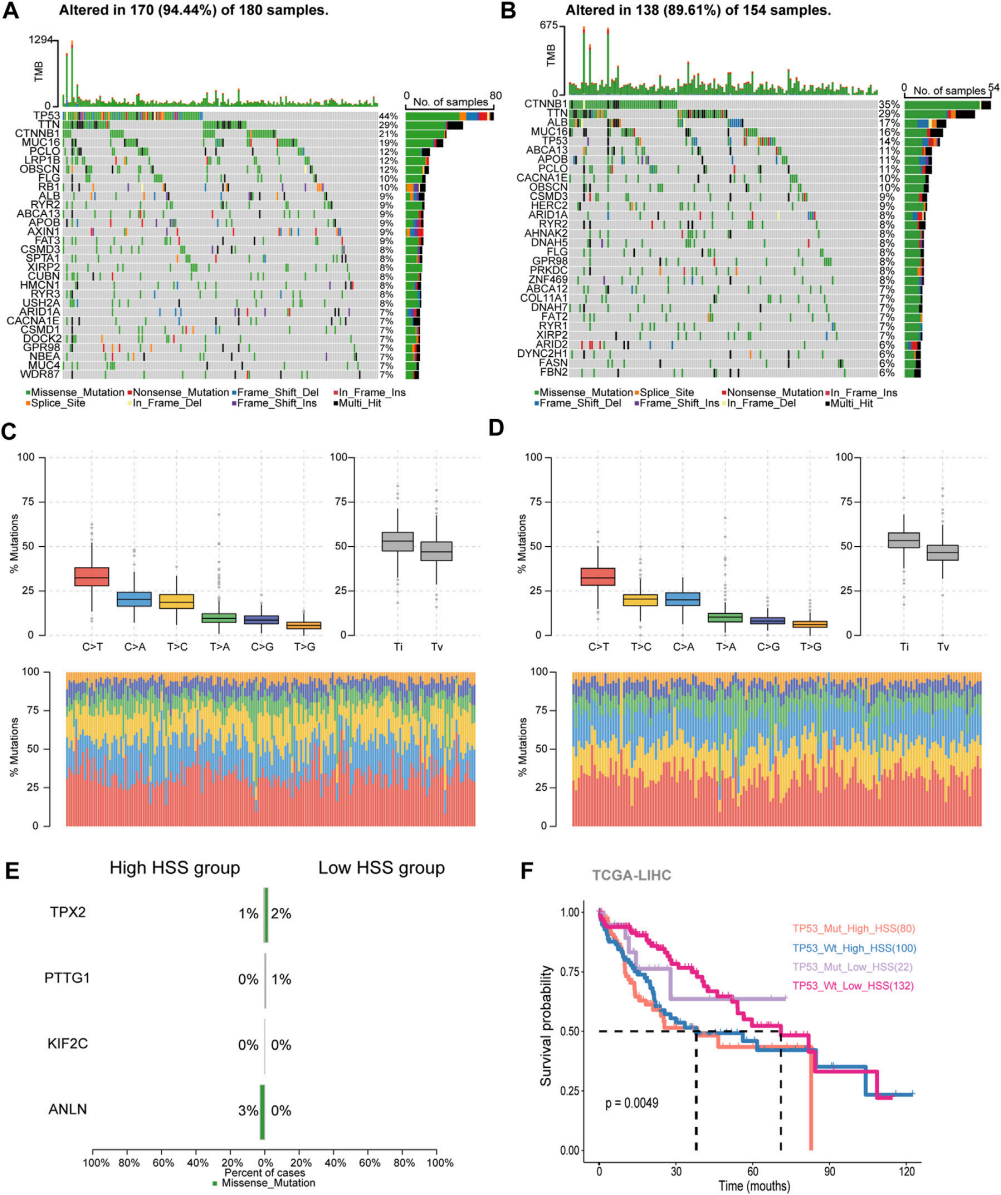
Study of Hepatocellular Carcinoma Mutation Spectrum. **(A)** Mutation map showing the frequency of alterations in the top 30 genes across TCGA-HSS-high samples. **(B)** Mutation map showing the frequency of alterations in the top 30 genes across TCGA-HSS-low samples. **(C, D)** The statistical results of Transitions_vs._Transversions for SNPs. **(E)** Mutation map of HS genes in the TCGA samples. **(F)** Kaplan–Meier curves based on High/Low HSS group and TP53 mutations in the TCGA-dataset-OS.

In the high HSS group, 94.5% of patients had gene mutations, with TP53 having the highest mutation frequency at 44% (Figure 3A). On the other hand, in the low HSS group, 89.6% of patients had mutations, with CTNNB1 having the highest mutation frequency. The mutation frequency of TP53 was only 14% (Figure 3B). Apart from these differences, we also found some commonalities; both high and low HSS groups were predominantly characterized by Missense_Mutation, and the results of single-nucleotide polymorphisms (SNPs) mostly involved variations between similar nucleotides (Figures 3C, D).

In terms of somatic mutation interactivity, we examined co-occurring or exclusive results between mutated genes. In the high HSS group, there was a significant co-mutation trend between TP53 and DOCK2 genes, while CTNNB1 showed a strong exclusive mutation tendency with AXIN1 (Supplementary Figure S3A). In the low HSS group, CTNNB1 showed co-mutation trends with several genes, including ARID2 and APOB, while TP53 also showed co-mutation trends with multiple genes (Supplementary Figure S3B). However, overall, both TP53 and CTNNB1 exhibited a tendency toward exclusive mutations in both datasets.

We also specifically examined the mutation status and tumor mutation burden of the four genes, but mutations in these four genes were not present in the majority of samples (Figure 3E). The results of tumor mutation burden showed that the low HSS group had a higher mutation burden (Supplementary Figure S3C, D). Finally, Finally, we reclassified the TCGA samples based on HSS grouping and TP53 mutations. we re-categorized the samples into four groups,TP53_Mut_High_HSS, TP53_Wt_High_HSS, TP53_Mut_Low_HSS, and TP53_Wt_Low_HSS.We supplemented our analysis by distinguishing between TP53 mutations and wild-type status within the High_HSS and Low_HSS groups. Survival analysis of these groups revealed that the TP53_Mut_High_HSS group exhibited the poorest survival outcomes (Figure 3F). This indicates that patients with both TP53 mutations and high HSS levels may face a particularly unfavorable prognosis. It underscores the limitation of relying solely on a transcriptional score model to predict prognosis accurately for all patient subtypes. Hence, considering the mutation status of patient genes in subsequent research is imperative and cannot be overlooked.

### 3.4 The connection between HSS and cells at the single-cell level

**FIGURE 4:**
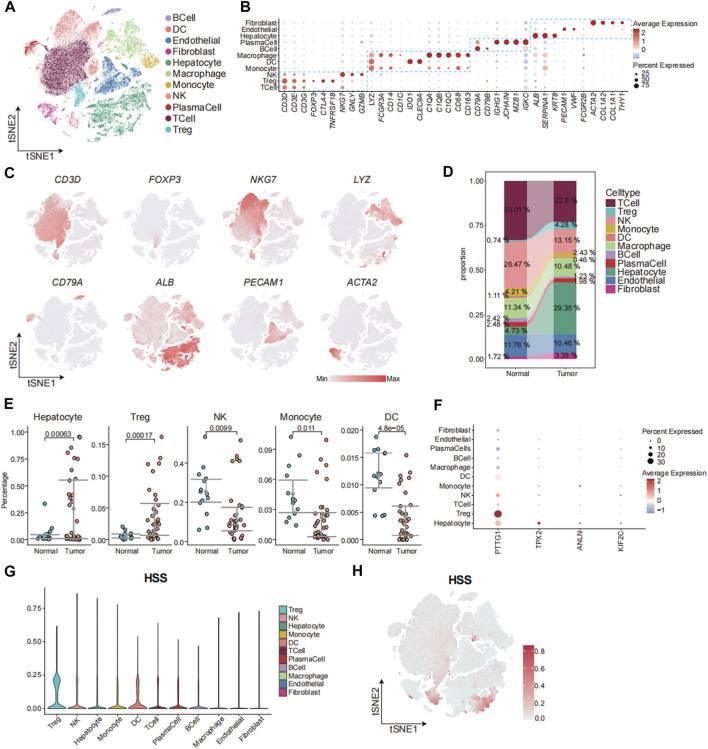
The Connection between HSS and Cells at the Single-Cell Level. **(A)** tSNE analysis identified 11 major cell types in hepatocellular carcinoma samples. **(B)** Dotplot showing the percentage of expressed cells and average expression levels of canonical marker genes of major cell types in cell clusters. **(C)** The tSNE plots showing the expression levels of signature genes of eight major cell types, colored by gene expression. **(D)** Sankey Diagram showing the percentages of 11 major cell types in Tissue sample. **(E)** Gittered scatter plot showing the percentages of cell types in each sample **(F)** Dotplot showing the percentage of expressed cells and average expression levels of HS marker genes in major cell types **(G)** Violin plot demonstrating HSS in 11 major cell types. **(H)** The tSNE plots showing the HSS of all cell types, colored by score levels.

Next, we aimed to elucidate the relationship between HSS and cells at the single-cell level. We obtained two sets of single-cell transcriptome data from hepatocellular carcinoma from the GEO database, namely, GSE149614 and GSE156625. Subsequently, rigorous quality control procedures were conducted, resulting in the acquisition of transcriptome data from 124,322 individual cells. We employed the Harmony algorithm to remove batch effects between samples and utilized an unsupervised clustering method based on shared nearest neighbors (SNN), identifying a total of 40 cell clusters (Supplementary Figure S4A-D). Cell clusters were annotated using canonical marker genes, followed by dimensionality reduction clustering and annotation into 11 major cell clusters, including T_Cell, Treg, NK_Cell, Monocyte, DC, Macrophage, B_Cell, and Plasma cells, Hepatocyte, Endothelial, and Fibroblast (Figure 4A-C; Supplementary Figure S4E). We observed an increased abundance of Hepatocyte, Fibroblast, and Treg populations in tumor tissues compared to adjacent normal tissues (Figure 4D, E; Supplementary Figure S4F). To further analyze the contribution of HSG to various cell types, we depicted the expression patterns of four genes across different cell types (Figure 4F). The results revealed significantly elevated expression of PTTG1 in Treg and Hepatocyte populations, with the expression of the other three genes also slightly higher in Hepatocytes compared to other cell types. Additionally, HSS demonstrated higher composite scores in Treg and select Hepatocyte populations (Figures 4G, H). In summary, leveraging single-cell transcriptome data, we found that HSS predominantly exhibited higher scores in Treg and Hepatocyte populations, emphasizing the association between HSG and Treg as well as Hepatocyte populations.

### 3.5 Transcriptome Analysis of Malignant Hepatocytes Based on HSS

**FIGURE 5:**
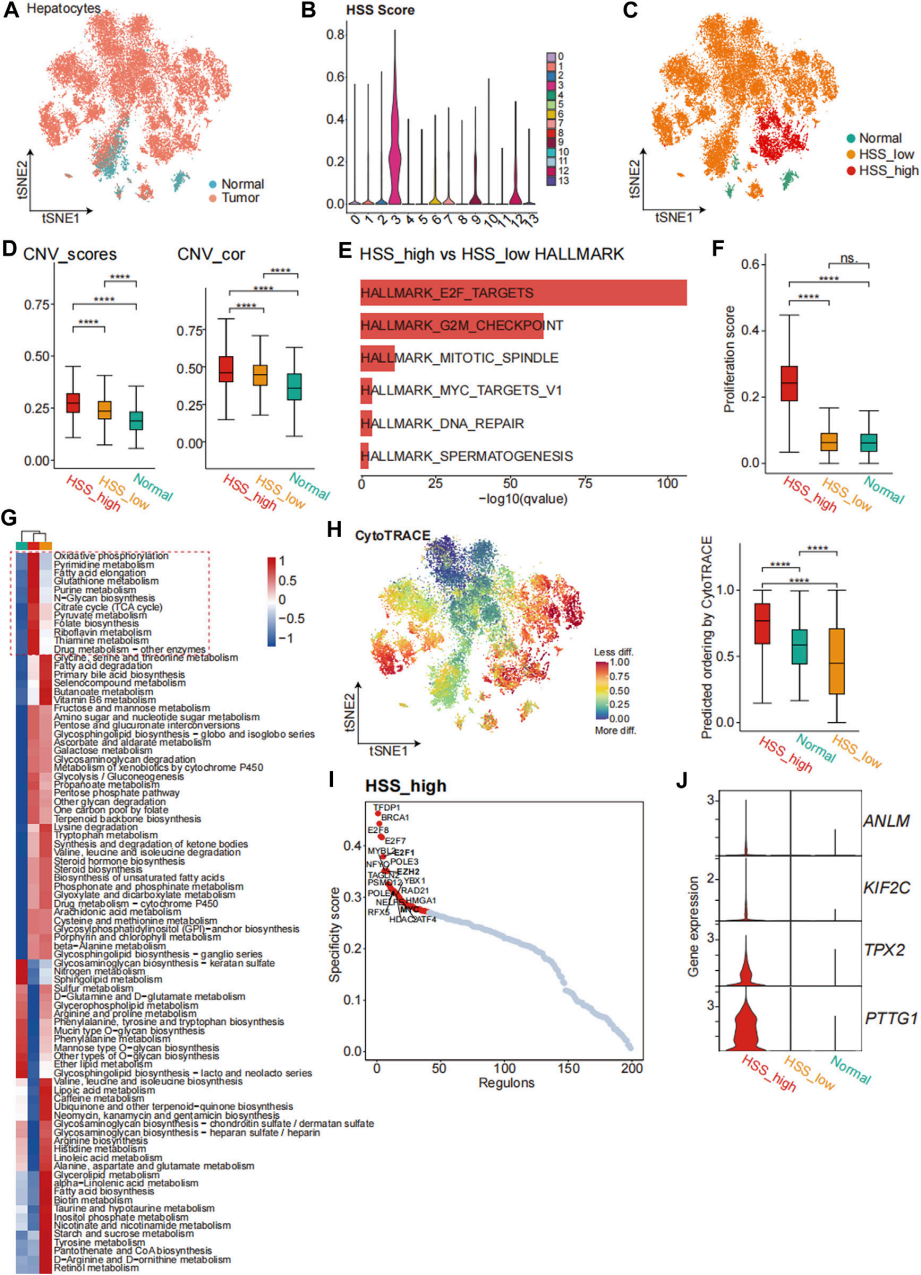
Transcriptome Analysis of Malignant Hepatocytes Based on HSS. **(A)** tSNE analysis shows the tissue distribution of Hepatocytes. **(B)** Violin plot demonstrating HSS in 14 clusters. **(C)** tSNE analysis identified 3 cell types in Hepatocyte. **(D)** Boxplot showing the HSS of 3 cell types in Hepatocyte. **(E)** Bar graph demonstrating the results of enrichment analysis of HALLMARK. **(F)** Boxplot demonstrating Proliferation score of 3 cell types in Hepatocyte. **(G)** scMetabolism-related cellular metabolic pathways. **(H)** CytoTRACE predicts the cell differentiation potential of 3 cell types in Hepatocyte. **(I)** Regulon analysis of pyscenic results. **(J)** Violin plot demonstrating HSG expression in 3 cell types of Hepatocyte.

To further analyze the relationship between hepatocytes and HSG, we separately extracted Hepatocytes and performed dimensionality reduction clustering of cell clusters, resulting in a total of 14 cell clusters (Supplementary Figure S5A). Subsequently, we considered cell clusters where tumor tissue comprised more than 50% of the cells as malignant hepatocytes. Accordingly, the 10th and 11th clusters were identified as normal hepatocytes (Figure 5A; Supplementary Figure S5B). Among the remaining cell clusters, analysis combined with HSS scores revealed that the third cluster had significantly higher scores compared to other cell clusters (Figure 5B; Supplementary Figure S5C). Finally, we categorized hepatocytes into three types, namely, Normal, HSS_high Hepatocyte, and HSS_low Hepatocyte (Figure 5C).

To ensure accurate identification of malignant tumors based on tissue origin, we utilized inferCNV to calculate the CNV scores of each cell. Additionally, we computed the correlation between CNV matrices of each cell and the top 10% of CNV matrices, revealing that HSS_high Hepatocyte had the highest CNV_scores and CNV_cor, followed by HSS_low Hepatocyte and Normal (Figure 5D). Scatter plots drawn based on the threshold defined by the second quartile also indicated that HSS_high Hepatocyte and Normal cells were mostly located in the first and third quadrants (Supplementary Figure S5D).

Malignant cells exhibited significant heterogeneity. We conducted differential expression analysis between HSS_high Hepatocyte and HSS_low Hepatocyte and found that genes upregulated in HSS_high Hepatocyte were enriched in pathways related to cell proliferation, such as the E2F pathway in HALLMARK analysis and cell cycle and cell senescence pathways in KEGG analysis. GO analysis also revealed enrichment in activities associated with cell division, corroborating results from bulk analysis (Figure 5E; Supplementary Figure S5E, F). Additionally, scoring using classic cell proliferation-related gene sets showed significantly higher scores in HSS_high Hepatocyte. Analysis of cell metabolism using scMetabolism also revealed increased activity of oxidative phosphorylation related to tumor proliferation in HSS_high Hepatocytes.

In addition to proliferative capacity, we predicted the differentiation potential of malignant cells using CytoTRACE, which suggested that HSS_high Hepatocytes may have higher differentiation potential (Figure 5H). Finally, using pyscenic, we inferred the transcription factors driving the stronger proliferative capacity and differentiation potential of HSS_high Hepatocyte. Results showed that the three groups of Hepatocytes exhibited different levels of activation for transcription factors. Interestingly, among the top 20 significantly activated transcription factors in HSS_high Hepatocyte, three (E2F1, EZH2, MYC) were found to potentially regulate PTTG1, which also exhibited higher gene expression levels in HSS_high Hepatocyte (Figure 5I; Supplementary Figure S5G). The activation scores and gene expression levels of transcription factors (E2F1, EZH2, MYC) were significantly higher in HSS_high Hepatocytes (Supplementary Figure S5H-I). Integration of transcription factors and PTTG1 in the STRING database also revealed significant associations among these proteins (Supplementary Figure S5K). In summary, analysis of hepatocellular carcinoma revealed that HSS_high Hepatocytes exhibited stronger proliferative capacity and differentiation potential, regulated by multiple transcription factors to upregulate PTTG1 expression.

### 3.6 Revealing the Impact of Key Gene PTTG1 on HCC Cell Phenotype and Treg Function

**FIGURE 6:**
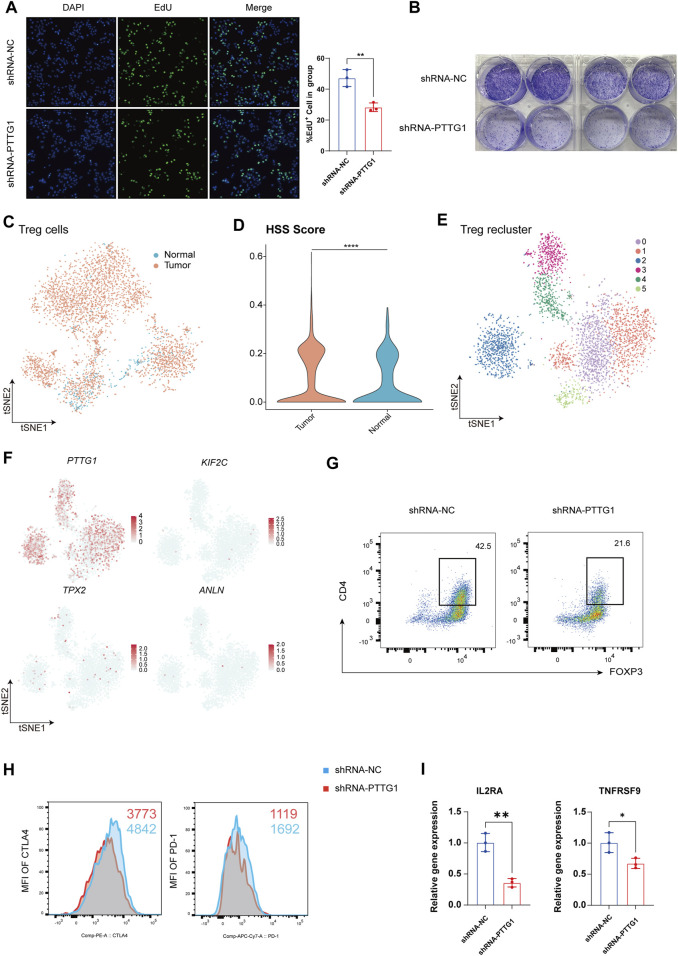
Revealing the Impact of Key Gene PTTG1 on HCC Cell Phenotype and Treg Function. **(A)** Representative images and counts of EdU^+^ cell in group of shRNA-NC and shRNA-PTTG1. **(B)** Examination of colony formation capabilities in group of shRNA-NC and shRNA-PTTG1. **(C)** tSNE analysis shows the tissue distribution of Treg. **(D)** Violin plot demonstrating HSS in Treg. **(E)** tSNE analysis identified 6 clusters in Treg. **(F)** The tSNE plots showing the expression levels of HSG in Treg, colored by gene expression. **(G)** Representative plots of the percentages of FOXP3^+^ cells in group of shRNA-NC and shRNA-PTTG1. **(H)** Representative plots of CTLA-4 and PD-1 expression in group of shRNA-NC and shRNA-PTTG1. **(I)** qPCR detection of IL2RA and TNFRSF9 in treated Treg; n = 3 per group. (*p < 0.05, **p < 0.01, ****p < 0.0001).

Next, We employed immunohistochemistry to detect the expression of PTTG1 in both cancerous and adjacent non-cancerous tissues of HCC patients, revealing a significant upregulation of PTTG1 expression within the cancerous tissues (Supplementary Figure S6A). Additionally, through the HPA database, we identified Treg as the immune cells exhibiting the highest expression of PTTG1, consistent with our research findings (Supplementary Figure S6B). Given the enhanced proliferative capacity and differentiation potential observed in HSS_high Hepatocyte, along with the significant differential expression of PTTG1 within this population, we identified PTTG1 as a pivotal gene for investigation. To explore the effects of PTTG1 on HCC cells, we utilized lentiviral knockdown of PTTG1 in the human hepatocellular carcinoma cell line Huh7 and assessed its cellular proliferative capacity. The results demonstrated a significant decrease in cell proliferation and colony formation ability in the shRNA-PTTG1 group compared to the shRNA-NC group (Figures 6A, B), suggesting an important role of PTTG1 in tumor proliferation.

Treg are crucial immune regulatory cells in the tumor microenvironment, playing a key role in maintaining an immune-suppressive environment within tumors (Li et al., 2020). Consistent with previous analyses, we found that besides its significant expression in hepatocytes, PTTG1 was also prominently expressed in Treg. Therefore, we conducted an analysis focusing on the role of PTTG1 in Treg. Treg was isolated for separate analysis, and in line with previous results, Treg exhibited a significant increase in quantity within tumor tissues (Figure 6C), with Treg in tumor tissues showing higher HSS scores (Figure 6D). Upon isolating Treg and performing dimensionality reduction clustering, a total of 6 cell clusters were identified (Figure 6E), and the distribution of HSG within each cluster was analyzed. The results revealed that among the four genes, only PTTG1 was extensively expressed in Treg (Figure 6F). To further investigate the impact of PTTG1 on Treg function, we knocked down PTTG1 in Treg using lentivirus. Compared to the shRNA-NC group, the shRNA-PTTG1 group exhibited a significant decrease in FOXP3 expression (Figure 6G). Additionally, crucial functional molecules PD-1 and CTLA4 also showed significant downregulation (Figure 6H). Subsequently, we endeavored to investigate the mechanisms by which PTTG1 influences Treg function. We classified Treg into PTTG1^high^ Treg and PTTG1^low^ Treg using single-cell data and conducted differential gene expression analysis and functional enrichment between these 2 cell populations (Supplementary Figure S6C-E). Our analysis revealed that compared to PTTG1^low^ Treg, PTTG1^high^ Treg exhibited elevated expression of FOXP3, consistent with our experimental results (Supplementary Figure S6F). Furthermore, we observed significant enrichment of the IL2_STAT5_SIGNALING in PTTG1^high^ Treg, a pathway closely associated with Treg function and stability (Supplementary Figure S6G) (Arenas-Ramirez et al., 2015). Among these, IL2RA and TNFRSF9 have been previously established as functional markers for tumor-infiltrating Treg (Cho et al., 2021; Permanyer et al., 2021). Subsequently, we assessed the expression levels of IL2RA and TNFRSF9 mRNA in the shRNA-PTTG1 group. Consistent with the analysis results, a significant reduction in the expression levels of IL2RA and TNFRSF9 in Treg following PTTG1 knockdown (Figure 6I). These findings indicate the significant role of PTTG1 in maintaining Treg function and establishing an immune-suppressive microenvironment within tumors.

### 3.7 Cell-cell interaction analysis

**FIGURE 7:**
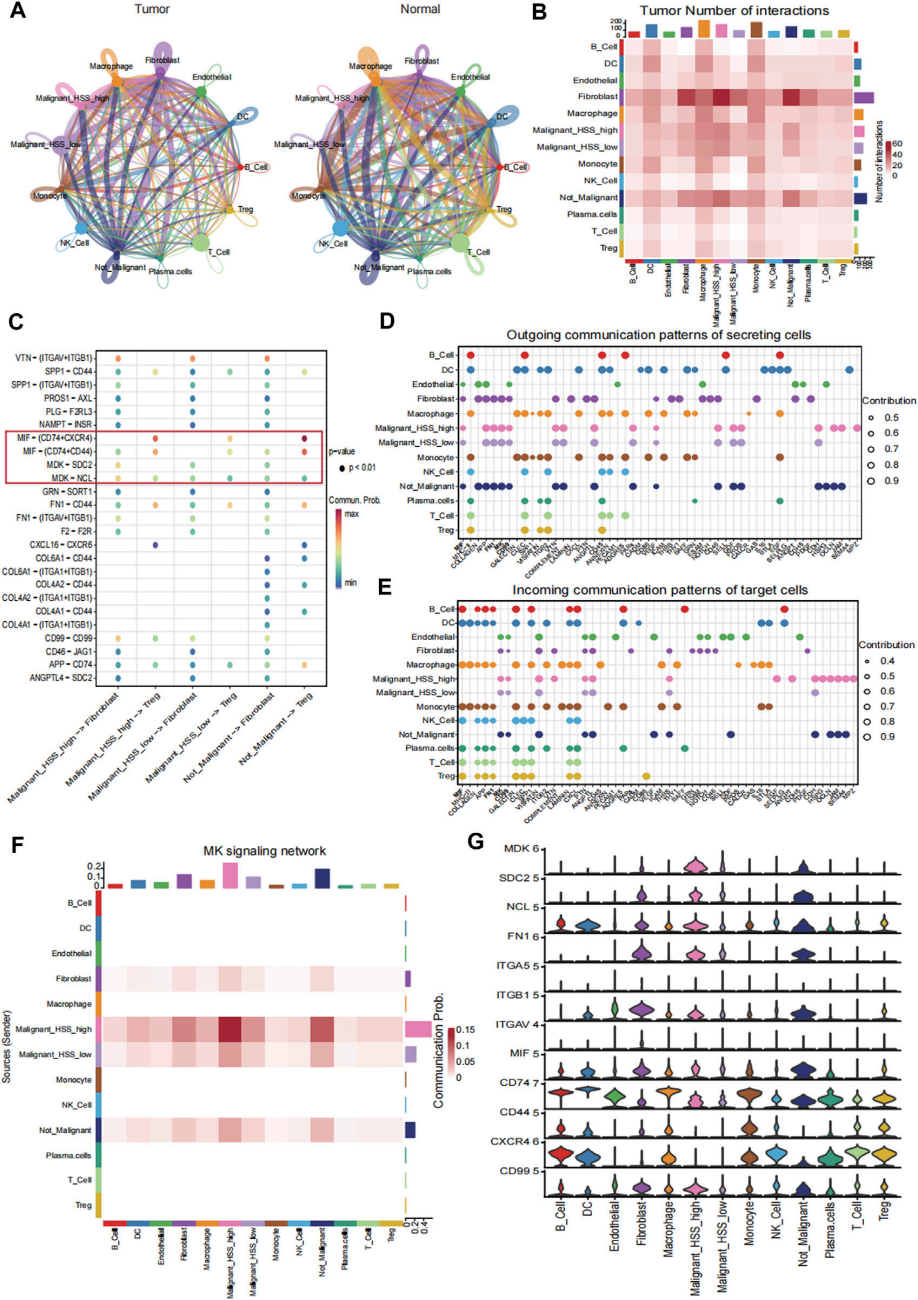
Cell-cell interaction analysis. **(A)** The interaction weights among all cell subpopulations in tumor and normal tissue. **(B)** The number of interactions among all cell subpopulations in tumor tissue. **(C)** Receptor ligand pairs of Malignant_HSS_high cells interacting with Fibroblasts and Treg. **(D, E)** The contribution values of outgoing or incoming signaling pathways for all cell groups indicate that the signaling pathway on the left side contributes the most. **(F)** The contribution of the MK signaling pathway to the interaction between Malignant_HSS_high cells and fibroblasts. **(G)** Violin plots showing the expression of genes associated with ligand-receptor pairs (**p < 0.01).

We performed CellChat analysis to identify key cell subpopulations and receptor-ligand pairs involved in interactions with Malignant_HSS_high cells in tumor and normal tissue. Initially, we explored the communication patterns among all cell subpopulations (Figure 7A). Our research findings indicate that fibroblasts are the most active communicating cell subtype in tumor tissues, and they also exhibit the highest interaction weights and number of interactions with Malignant_HSS_high cells among all cell types (Figures 7A, B). In contrast, macrophages in normal tissues demonstrate more active cell communication (Figure 7A; Supplementary Figure S7A). In our specific ligand-receptor interaction analysis, we primarily focused on fibroblasts. However, due to the significant expression of PTTG1 in both Malignant_HSS_high cells and Treg, we also considered Treg. When Malignant_HSS_high cells as ligands, the interactions with fibroblasts involve MDK-SDC2/NCL, while interactions with Treg involve MIF-CD44/CXCR4/CD74 (Figure 7C). The Malignant_HSS_high cells as receptors, significantly different ligand-receptor pairs include FN1-ITGAV/ITGB1ITGA5 and CD99^−^CD99 (Supplementary Figure S7B, C).

These findings are also validated in the analysis of active communication pathways. For example, the MK pathway not only ranks ahead of many pathways but also contributes most significantly to the interactions between malignant cells and fibroblasts (Figures 7D–F). Lastly, we corroborated the interactions by examining the expression levels of the genes involved in these ligand-receptor pairs (Figure 7G).

## 4 Discussion

The aging process is a continuous and unavoidable occurrence in the human body, participating in various physiological and pathological processes (Campisi, 2013). Several diseases have been linked to cellular senescence in previous research, including atherosclerosis (Gardner et al., 2015), diabetes (Palmer et al., 2019), NAFLD (Ogrodnik et al., 2017), and others. The relationship between senescence and tumors is complex. On one hand, aging serves as one of the body’s protective mechanisms against tumor development. For example, the activation of oncogenes can induce cellular senescence, limiting tumor growth and preventing benign tumors from progressing to malignancy (Courtois-Cox et al., 2008). At the same time, senescence of tumor cells can induce a senescence-associated secretory phenotype (SASP), leading to the secretion of pro-inflammatory cytokines such as IL-1, IL-6, IL-8, which triggers an immune response and promotes the clearance of tumor cells (Chibaya et al., 2022). However, from another perspective, the accumulation of senescent cells with age creates a microenvironment conducive to tumor development. This includes the decline in immune function due to the aging of immune cells, as well as the senescence-associated secretory phenotype (SASP) resulting from fibroblast senescence, which contains factors such as MMP3 and VEGF that can promote tumor growth (Krtolica et al., 2001; Liu and Hornsby, 2007). Not only age-related factors, but tumors themselves can also promote their growth by inducing senescence in surrounding cells. Additionally, chemotherapy-induced cellular senescence is increasingly recognized as one of the reasons for the rapid emergence of drug resistance in malignant tumors (Xu et al., 2019). Understanding the role of aging in tumors and identifying the distribution and changes in the expression of aging-related genes in the tumor environment is crucial for effectively utilizing cellular senescence in the treatment of cancer.

In this study, we conducted a comprehensive analysis of four independent datasets from HCCDB v2.0 and constructed a prognostic risk model containing four stable HSG, scoring the risk accordingly. We found that the high HSS group exhibited significantly worse prognosis, along with higher tumor purity, more active cell proliferation, and increased infiltration of immunosuppressive Treg. This result confirms the relevance of HSG to HCC and underscores the credibility of predicting HCC prognosis through HSG.

Subsequently, using single-cell data, we further elucidated the relationship between HSS and HCC. We found that HSS had the highest scores in Treg and Hepatocyte, with HSS_high Hepatocyte exhibiting stronger proliferative capacity and differentiation potential. Interestingly, among the four HSG, PTTG1 showed the most significant role, manifested not only in widespread high expression but also in the upregulation of its associated transcription factors. PTTG1 is involved in DNA damage repair regulation as well as organ development and metabolism. Its high expression in various endocrine-related tumors is associated with the process of tumor metastasis (Vlotides et al., 2007). In HCC, PTTG1 has been shown to promote tumorigenesis by influencing asparagine metabolism, and our experiments have confirmed that PTTG1 can enhance tumor cell proliferation (Zhou et al., 2023). Specifically, we found that PTTG1 is associated with the function of Treg. Knocking down PTTG1 reduces the induction of Foxp3^+^ Treg and decreases the expression of functional molecules CTLA-4 and PD-1. This aligns with the increased Treg infiltration observed in the high HSS group. Here, we have conducted an initial exploration into the mechanism by which PTTG1 influences Treg function. Our findings suggest that PTTG1 may be involved in the regulation of the IL2-STAT5 signaling pathway in Treg. This pathway plays a crucial role in maintaining the stability of Treg function. However, the role and specific mechanisms of PTTG1 in Treg still need to be further explored, which may help in developing personalized treatment plans for HCC patients based on HSS.

At last, through intercellular communication analysis, we found that fibroblasts are the most active cell subtype in tumor tissue communication, and they have various receptors with HSS_high cells, indicating their potential relevance to the appearance of HSS_high cells and playing an important role, which requires further investigation.

## 5 Conclusion

Our study comprehensively reveals the significant role of HSG in the development of HCC, as well as its intricate relationships with the immune microenvironment, mutational burden, and single-cell level cells. In particular, the role of PTTG1 in Treg and HCC cells is highlighted. These findings provide important clues for a deeper understanding of the molecular mechanisms underlying HCC and the development of prognostic prediction models.

## Data Availability

The Bulk RNA-seq data presented in the study are deposited in the HCCDB repository, accession number HCCDB15, HCCDB18, HCCDB25, HCCDB30, via the link http://lifeome.net:809/#/repository?type=buke. The scRNA-seq data presented in the study are deposited in the GEO repository, accession number GSE149614, GSE156625.
