# Corrigendum: Integrated analysis of single-cell and bulk RNA sequencing data reveals a cellular senescence-related signature in hepatocellular carcinoma

**DOI:** 10.3389/fcell.2024.1519902

**Published:** 2024-11-12

**Authors:** Lei Qiao, Zibo Xu, Yuheng Chen, Wenwei Chen, Yuan Liang, Yi Wei, Kang Wang, Yue Yu, Wei Yan

**Affiliations:** ^1^ Hepatobiliary Center, The First Affiliated Hospital of Nanjing Medical University, Key Laboratory of Liver Transplantation, Chinese Academy of Medical Sciences, NHC Key Laboratory of Living Donor Liver Transplantation (Nanjing Medical University), Jiangsu Provincial Medical Innovation Center, Jiangsu Provincial Medical Key Laboratory, Nanjing, Jiangsu Province, China; ^2^ Department of Bioinformatics, Nanjing Medical University, Nanjing, China; ^3^ School of Public Health, Southeast University, Nanjing, Jiangsu Province, China; ^4^ School of Biological Science and Medical Engineering, Southeast University, Nanjing, Jiangsu Province, China; ^5^ Department of Burn and Plastic Surgery, The First Affiliated Hospital of Nanjing Medical University, Nanjing, Jiangsu Province, China

**Keywords:** cellular senescence, hepatocellular carcinoma, prognosis, tumor immune microenvironment, treg

In the published article, there was an error in the **Funding** statement. The NSFC funding number was incorrect; the number “82270506” should instead be “82270607.” The correct Funding statement appears below.

