# Supplementary material for: Integrated analysis of single-cell and bulk RNA sequencing data reveals a cellular senescence-related signature in hepatocellular carcinoma
Source: Front Cell Dev Biol. 2024 Jun 3;12:1407428. doi: 10.3389/fcell.2024.1407428 (PMC11180799; doi:10.3389/fcell.2024.1407428)
Supplement: Supplementary file 2 [file Table1.DOCX]

| **Characteristic** | **Total[756]** | **GSE148355[48(6.3%)]** | **ICGC[203(27%)]** | **OEP000321[158(21%)]** | **TCGA[347(46%)]** |
| --- | --- | --- | --- | --- | --- |
| **Gender** |  |  |  |  |  |
| **Female** | **194 (26%)** | **7 (15%)** | **50 (25%)** | **30 (19%)** | **112 (32%)** |
| **Male** | **557 (74%)** | **41 (85%)** | **153 (75%)** | **128 (81%)** | **235 (68%)** |
| **Age** |  |  |  |  |  |
| **Mean (SD)** | **60 (13)** | **55 (10)** | **67 (10)** | **54 (11)** | **60 (13)** |
| **Median (IQR)** | **61 (52, 69)** | **55 (49, 60)** | **69 (62, 74)** | **54 (46, 62)** | **61 (52, 69)** |
| **Range** | **17, 90** | **36, 78** | **31, 86** | **20, 81** | **17, 90** |
| **Stage** |  |  |  |  |  |
| **Other** | **24 (3.2%)** | **0 (0%)** | **0 (0%)** | **0 (0%)** | **24 (6.9%)** |
| **Stage I** | **301 (40%)** | **15 (31%)** | **33 (16%)** | **90 (57%)** | **163 (47%)** |
| **Stage II** | **209 (28%)** | **21 (44%)** | **96 (47%)** | **14 (8.9%)** | **78 (22%)** |
| **Stage III** | **199 (26%)** | **9 (19%)** | **59 (29%)** | **52 (33%)** | **79 (23%)** |
| **Stage IV** | **23 (3.0%)** | **3 (6.3%)** | **15 (7.4%)** | **2 (1.3%)** | **3 (0.9%)** |
| **Status** |  |  |  |  |  |
| **Alive** | **531 (70%)** | **42 (88%)** | **168 (83%)** | **102 (65%)** | **219 (63%)** |
| **Dead** | **225 (30%)** | **6 (13%)** | **35 (17%)** | **56 (35%)** | **128 (37%)** |
| **Virus** |  |  |  |  |  |
| **HBV** | **180 (24%)** | **36 (75%)** | **53 (26%)** | **0 (0%)** | **91 (26%)** |
| **HBV,HCV** | **11 (1.5%)** | **0 (0%)** | **4 (2.0%)** | **0 (0%)** | **7 (2.1%)** |
| **HCV** | **168 (22%)** | **5 (10%)** | **117 (58%)** | **0 (0%)** | **46 (13%)** |
| **Other** | **397 (52%)** | **7 (15%)** | **29 (14%)** | **158 (100%)** | **203 (58%)** |

**HBV, hepatitis B virus; HCV, hepatitis C virus.**
