# Supplementary material for: Integrated analysis of single-cell and bulk RNA sequencing data reveals a cellular senescence-related signature in hepatocellular carcinoma
Source: Front Cell Dev Biol. 2024 Jun 3;12:1407428. doi: 10.3389/fcell.2024.1407428 (PMC11180799; doi:10.3389/fcell.2024.1407428)
Supplement: Supplementary file 3 [file DataSheet1.docx]

Supplementary Material

Integrated analysis of single-cell and bulk RNA sequencing data reveals a cellular senescence-related signature in hepatocellular carcinoma

Lei Qiao, Zibo Xu, Yuheng Chen, Wenwei Chen, [Yuan Liang](https://pubmed.ncbi.nlm.nih.gov/?term=Liang+Y&cauthor_id=37994257), Yi Wei, Kang Wang, Yue Yu, Wei Yan

*** Correspondence:** Wei Yan [yivigg@163.com;](mailto:yivigg@163.com;) Yue Yu yuyue@njmu.edu.cn

# Supplementary Figures and Tables

## Supplementary Figures

**
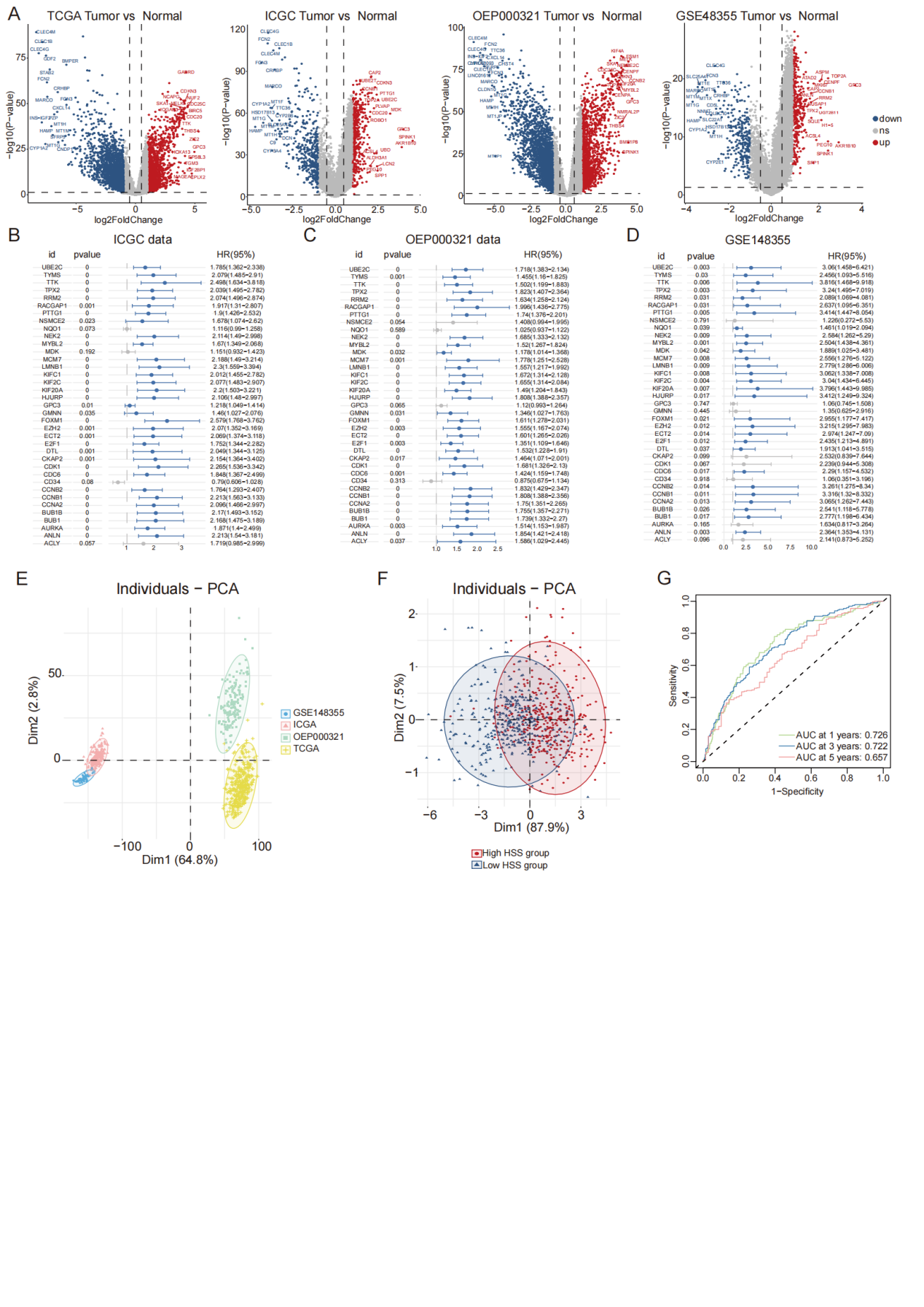
**

**Supplementary Figure 1 Prognostic Biomarkers and Risk Assessment in Hepatocellular Carcinoma.** (A) Differential expression analysis of Tumor samples and adjacent normal tissues samples in TCGA, ICGC, OEP000321, GSE148355 (p-value< 0.05 and log2FC > 1).(B-D) Univariate Cox regression of differential genes for the screening of prognosis-related genes in ICGC, OEP000321, GSE148355 cohort. (E) Principal component analysis (PCA) of the 4 separate datasets. (F) Principal component analysis (PCA) of the High/Low HSS group. (G) Results of time-dependent ROC curves for the risk model in the Integrated dataset.、

**
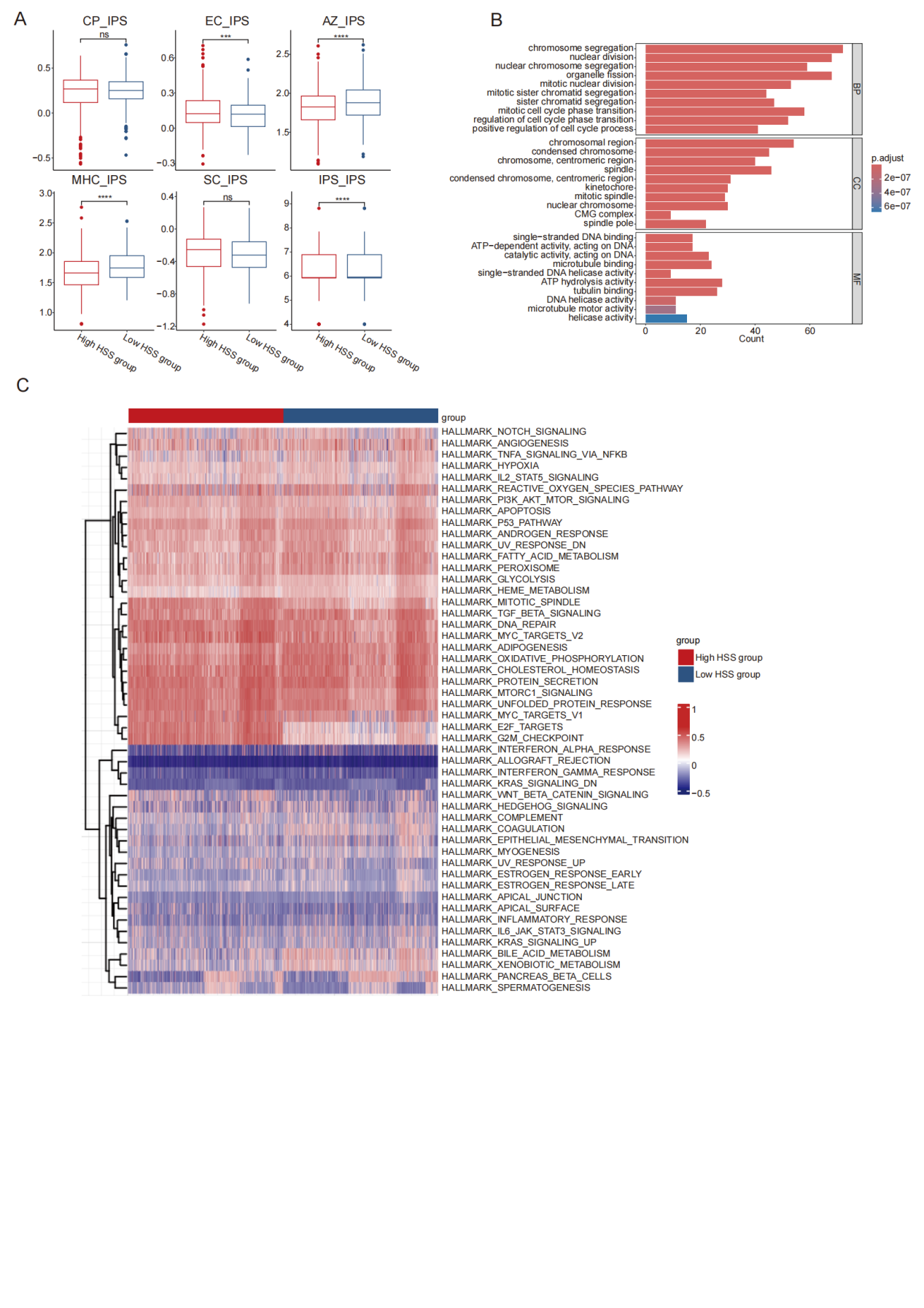
**

**Supplementary Figure2 Analysis of Immune Status and Function in High and Low HSS Groups.** (A) Immunophenoscore of two groups in the Integrated dataset. (B) Gene Ontology (GO) enrichment analysis of the up signature genes in High HSS group. (C) Heatmap of HALLMARK pathways.


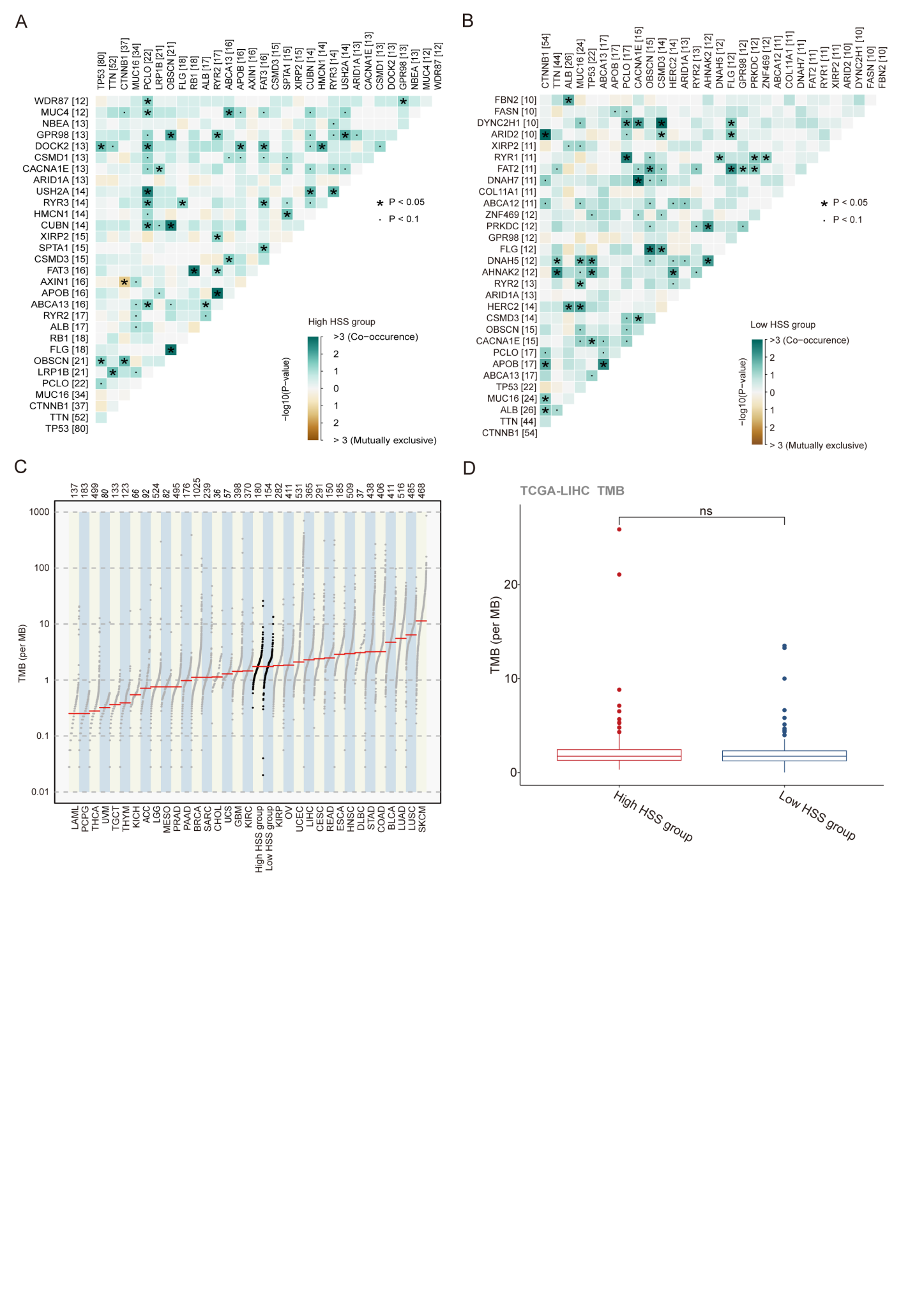


**Supplementary Figure3 Analysis of Somatic Mutations and Tumor Mutational Burden in Cancer Subtypes and High/Low HSS Groups.** (A-B) The co-occurring and exclusiveness among somatic mutations.(C)The tumor mutational burden across 33 types of cancer(D)The tumor mutational burden in high HSS and low HSS groups.


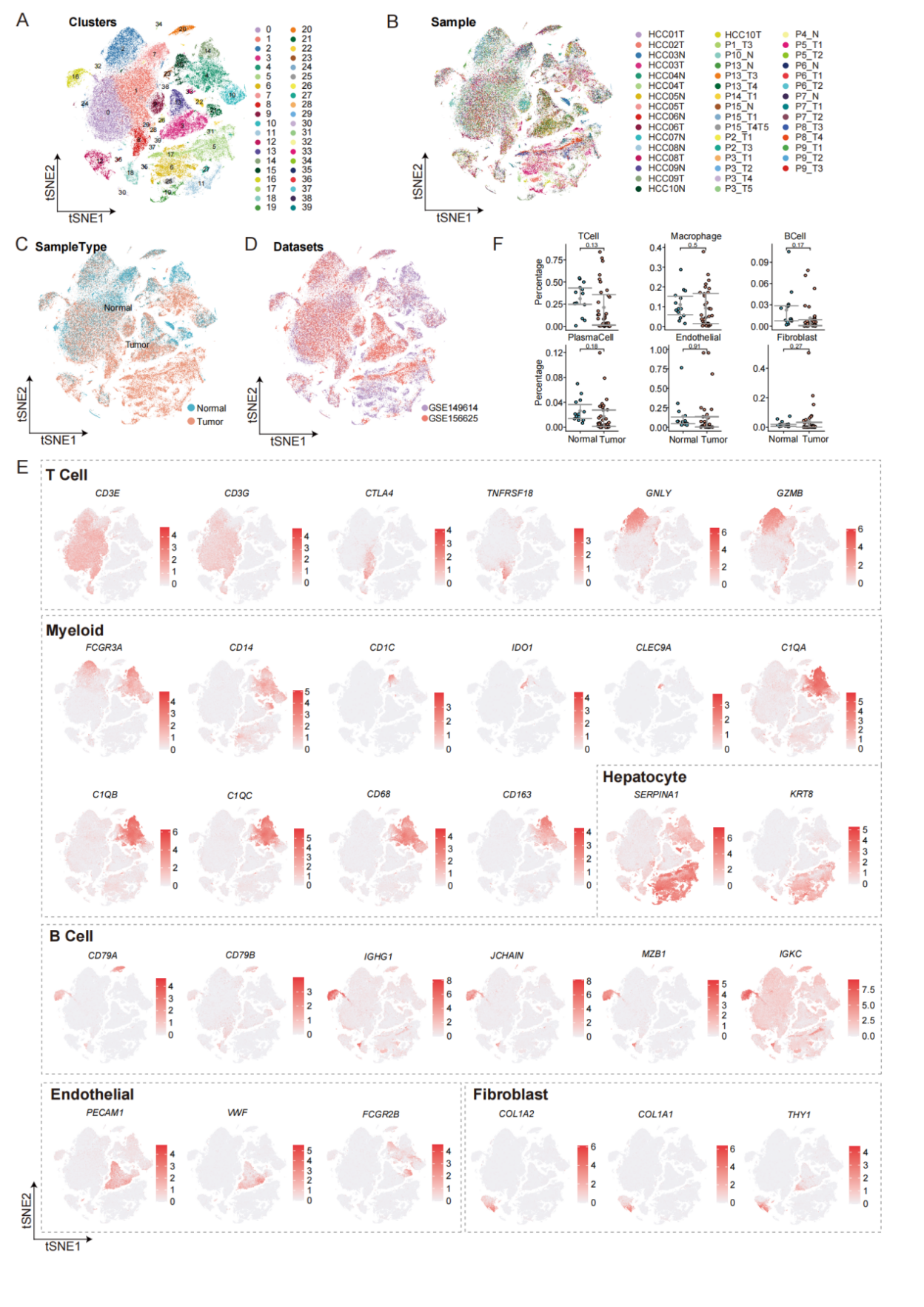


**Supplementary Figure4 Heterogeneous Cell Subpopulations and Sample Distributions in Hepatocellular Carcinoma.** (A) tSNE analysis identified 40 clusters in hepatocellular carcinoma samples. (B) tSNE analysis shows the sample distribution of hepatocellular carcinoma. (C) tSNE analysis shows the tissue distribution of hepatocellular carcinoma. (D) tSNE analysis shows the dataset distribution of hepatocellular carcinoma. (E) The tSNE plots showing the expression levels of signature genes of 6 major cell types, colored by gene expression. (F) Gittered scatter plot showing the percentages of cell types in each sample.


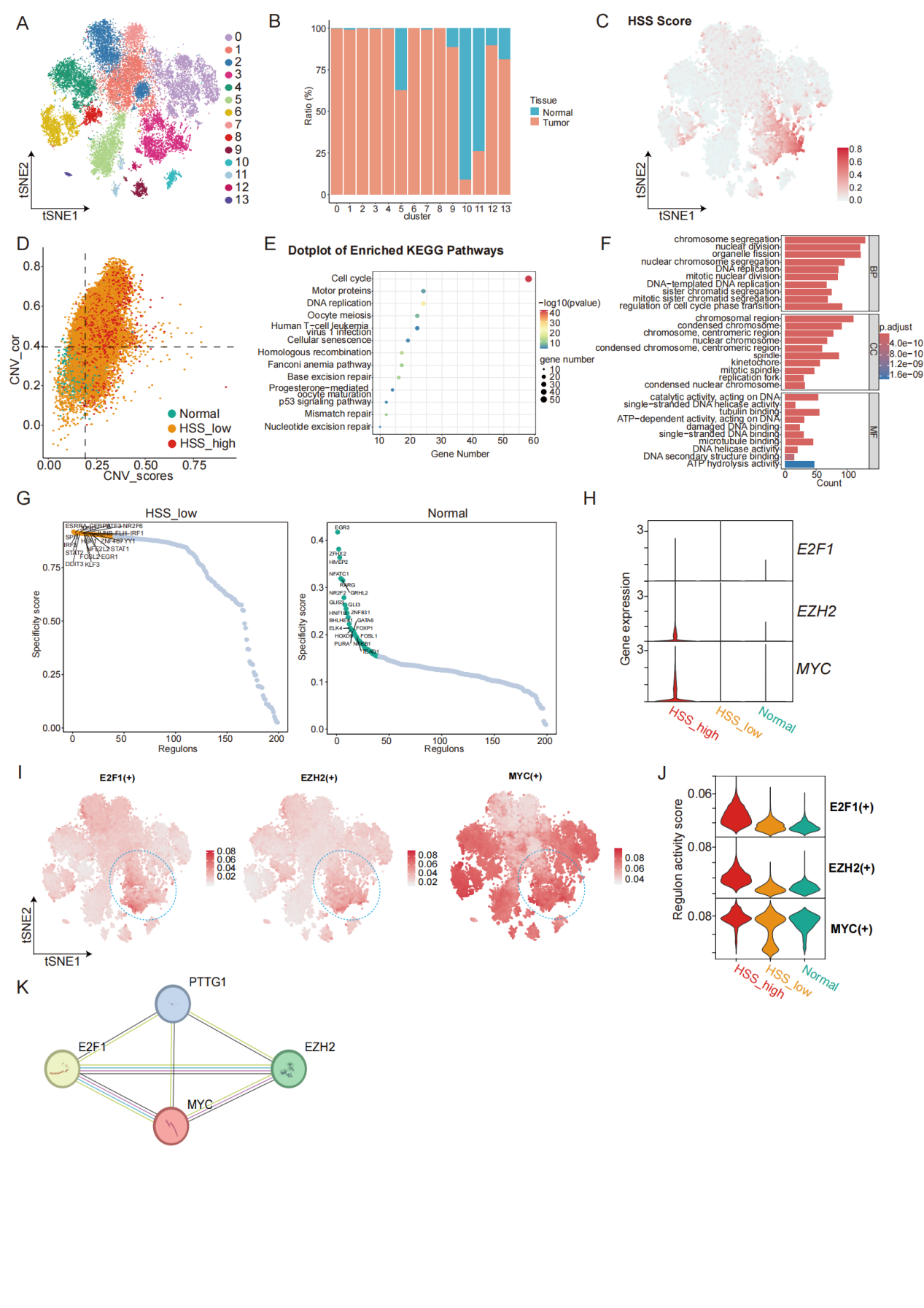


**Supplementary Figure5 Transcriptome Analysis of Malignant Hepatocytes Based on HSS.** (A) tSNE analysis identified 14 clusters in Hepatocyte. (B) Bar graph demonstrating the percentages of Tissue in each cluster. (C) The tSNE plots showing the HSS of all Hepatocyte, colored by score levels. (D) Scatter plot showing distribution of 3 cell types in Hepatocyte. (E) KEGG enrichment analysis. (F) Gene Ontology (GO) enrichment analysis. (G) Regulon analysis of the PySCENIC results for HSS_low and Normal. (H) Violin plot demonstrating TF genes expression in 3 cell types of Hepatocyte. (I) The tSNE plots showing the Regulon activity score of TF of all Hepatocyte, colored by score levels. (J) Violin plot demonstrating the Regulon activity score of TF in 3 cell types of Hepatocyte. (K) The protein interaction network from STRING.


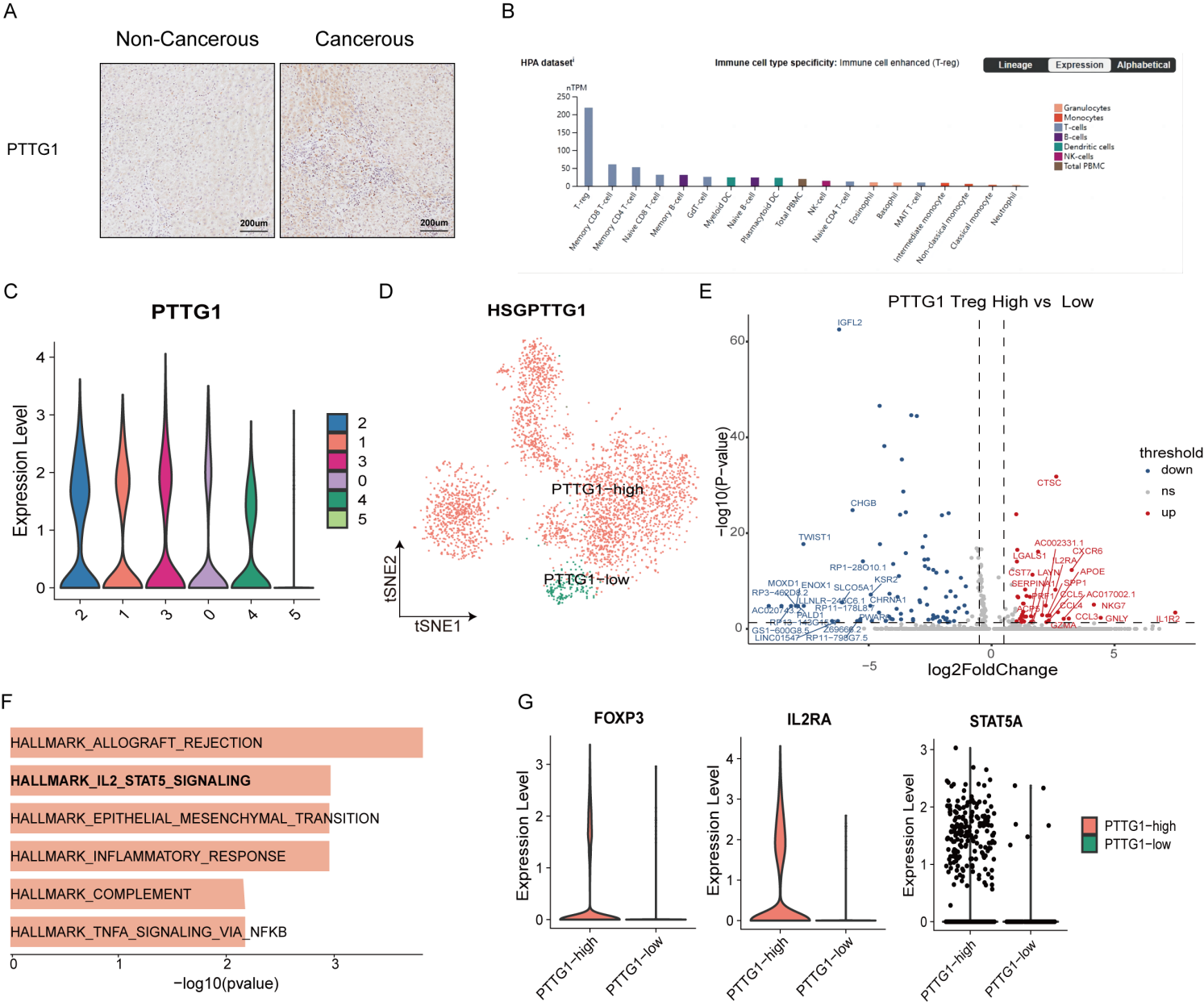


**Supplementary Figure6 Characterization of PTTG1 Expression and Its Impact on Treg Subpopulations.** (A) Immunohistochemical staining of PTTG1 on cancerous and adjacent non-cancerous tissues; Scale bar, 200 um. (B) The expression of PTTG1 in different immune cells from the HPA database. (C) Violin plot showing PTTG1 in 6 subgroups of Treg. (D) The tSNE plot showing the distribution of PTTG1high and PTTG1low populations within Treg. (E) Differential expression analysis of PTTG1high group and PTTG1low Group. (p-value< 0.05 and log2FC > 1). (F) Bar graph demonstrating the results of enrichment analysis of HALLMARK. (G) Violin plot showing expression of FOXP3,IL2RA and STAT5A in PTTG1^high^ group and PTTG1^low^ Group.


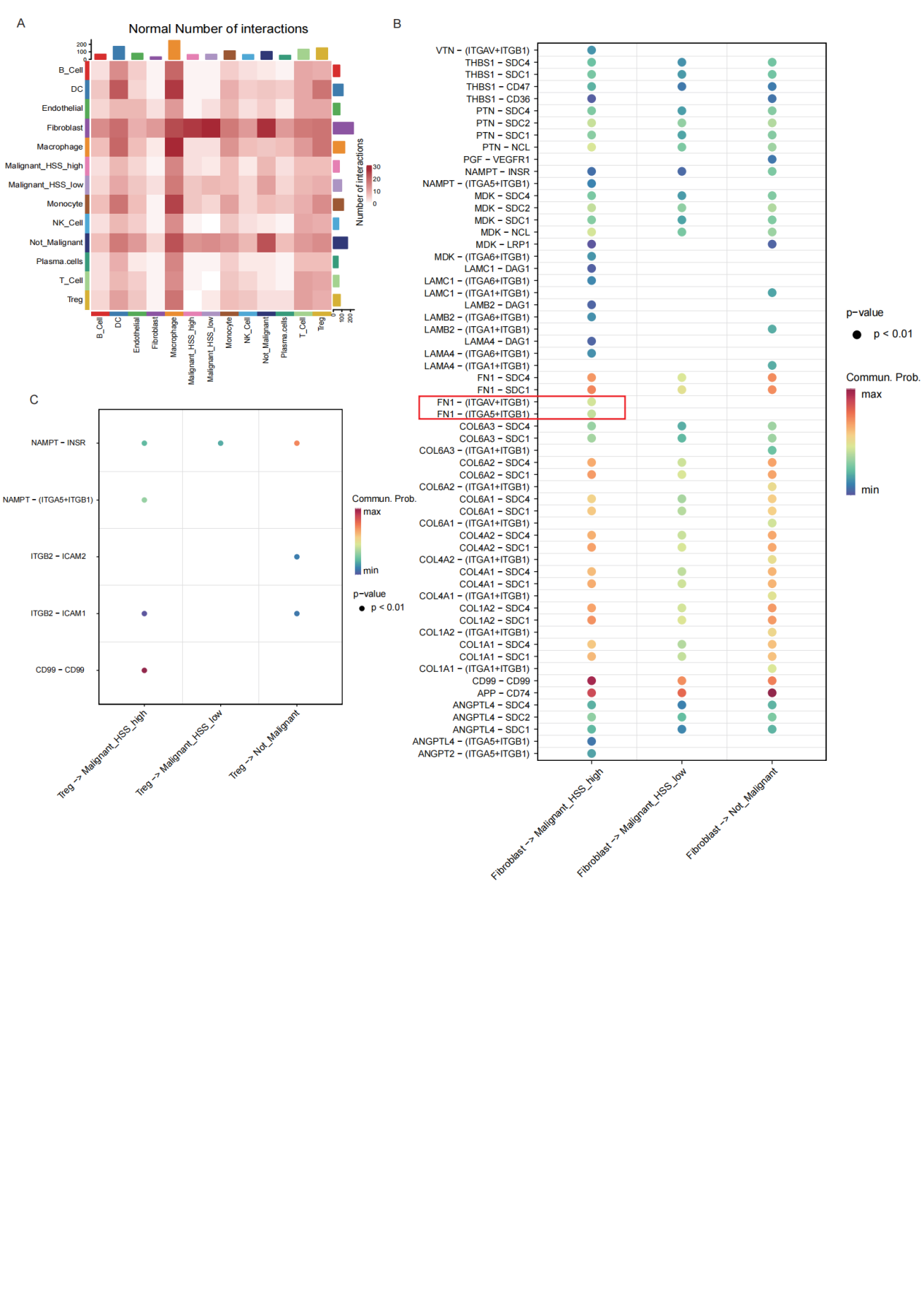


**Supplementary Figure7 Interactions between Cell Subpopulations in Normal Tissue and Malignant Cells with Fibroblasts and Treg.** (A) The number of interactions among all cell subpopulations in normal tissue. (B-C) Receptorligand pairs of Malignant_HSS_high cells interacting with Fibroblasts and Treg.

## Supplementary Tables

use excel sheets to submit the supplemental tables.

Supplementary table S1：Data Resource

Supplementary table S2：cellular senescence marker

Supplementary table S3： cell proliferation gene set

Supplementary table S4： primer
